# The maximum chemiluminescence intensity predicts severe neutropenia in gemcitabine-treated patients with pancreatic or biliary tract cancer

**DOI:** 10.1007/s00280-018-3685-6

**Published:** 2018-09-14

**Authors:** Koki Goto, Ryusei Matsuyama, Yusuke Suwa, Sayaka Arisaka, Toshiaki Kadokura, Mari Sato, Ryutaro Mori, Takafumi Kumamoto, Masataka Taguri, Itaru Endo

**Affiliations:** 1 Department of Gastroenterological Surgery, Yokohama City University Graduate School of Medicine, 3-9 Fukuura, Kanazawa-ku, Yokohama, 236-0004 Japan; 2 Department of Biostastics and Epidemiology, Yokohama City University Graduate School of Medicine, Yokohama, Japan

**Keywords:** Biliary tract cancer, Chemiluminescence, Gencitabine, Neoadjuvant chemotherapy, Neutropenia, Pancreatic cancer

## Abstract

**Purpose:**

To assess the predictive ability of the maximum chemiluminescence intensity (CI_max_) for severe neutropenia (SN) during neoadjuvant chemo(radio)therapy [NAC(RT)] in patients with advanced pancreatic or biliary tract cancer.

**Methods:**

Clinicopathological variables and blood test data before NAC(RT) were evaluated in 64 patients with advanced pancreatic or biliary tract cancer who received gemcitabine plus tegafur/gimeracil/oteracil as NAC(RT).

**Results:**

Thirty-nine patients (60.9%) developed Grade 3–4 SN. The median time between commencing NAC(RT) and the onset of SN was 15 (range 10–36) days. SN occurred during the NAC period, not the RT period. The CI_max_, neutrophil count, serum interleukin-6 level, C-reactive protein level, complement C3 titer, serum complement titer, and 50.0% hemolytic unit of complement before NAC(RT) were significantly lower in patients with SN than in those without SN (*P* < 0.05). Multivariate analysis confirmed the CI_max_ to be the sole independent predictor of SN (*P* < 0.05). The optimal threshold for the CI_max_ was 46,000 RLU/s. The sensitivity and specificity were 46.2% and 80.0%, respectively. Majority of the patients (81.8%) with a low CI_max_ before NAC(RT) experienced SN during NAC(RT).

**Conclusions:**

CI_max_ before NAC(RT) predicts SN during NAC(RT) in patients with advanced pancreatic or biliary tract cancer.

## Introduction

Majority of the patients with advanced pancreatic or biliary tract cancer have a poor prognosis. Complete surgical resection is currently the only potentially curative treatment for long-term survival. However, majority of the patients considered to have localized cancer by radiographic examination actually have undetected systemic disease and are unlikely to benefit from surgical treatment alone [1, 2]. However, with rapid developments in chemotherapeutic regimens, both adjuvant chemotherapy and neoadjuvant chemo(radio)therapy (NAC(RT)) have been shown to be beneficial for patients with borderline resectable pancreatic cancer [3] or advanced biliary tract cancer [4]. While gemcitabine plus tegafur/gimeracil/oteracil (GS) as NAC(RT) has been reported to be safe and effective for patients with borderline resectable pancreatic cancer [5], GS as adjuvant chemotherapy [6] and gemcitabine as NAC [7] have been reported to be safe and effective for patients with advanced biliary tract cancer.

We have performed NAC(RT)-GS in patients with advanced pancreatic or biliary tract cancer. Bone marrow suppression, an adverse effect of anticancer drugs, was frequently observed in patients treated with NAC(RT)-GS. The incidence of severe neutropenia (SN) [Grade ≥ 3 according to the Common Terminology Criteria for Adverse Events (version 4.0) [8]] was particularly high and has been reported in approximately 62.2% of patients [9]. SN is a major toxicity that forces a reduction in the relative dose intensity (RDI) of the anticancer drugs used in NAC(RT). SN during NAC(RT) can also complicate tumor resection. However, the risk of SN during gemcitabine-based therapy has not been extensively studied.

Previous studies have demonstrated that risk factors for SN and febrile neutropenia include old age [10, 11], female sex [12], a poor Eastern Cooperative Oncology Group performance status [13], a low body mass index [14], a small body surface area [15], a history of cardiovascular disease [10], diabetes mellitus [16], a poor nutritional status, inflammation [11, 13], and a low baseline absolute neutrophil count (ANC) [17]. Kiguchi et al. [18] reported that the maximum chemiluminescence intensity (CI_max_), as assessed by an in vitro reaction between peripheral neutrophils and endotoxin, is indicative of the maximum neutrophil activity in whole blood. A low CI_max_ is also associated with the exhaustion of peripheral polymorphonuclear leukocytes. CI_max_ has been suggested to be predictive of mortality in patients with sepsis. Therefore, in addition to the baseline ANC, we also focused on neutrophil activity for predicting the onset of SN.

The aim of this study was to investigate potential markers of SN in patients with advanced pancreatic or biliary tract cancer who received NAC(RT)-GS by evaluating clinicopathological variables and nutritional and immune markers (including CI_max_) before NAC(RT).

## Materials and methods

### Study population

We conducted a retrospective observational study in the Department of Gastroenterological Surgery at Yokohama City University Graduate School of Medicine (Yokohama, Japan). The study protocol was approved by the Ethical Review Board of Yokohama City University Hospital (Yokohama, Japan) (approval number: 121101023). Sixty-four chemo-naïve patients with histologically proven advanced pancreatic or biliary tract cancer who were treated with NAC(RT)-GS between June 2013 and December 2015 were analyzed. Patients with multiple primary cancers and a history of prior chemotherapy, as well as those who did not complete NAC(RT)-GS, were excluded.

Pancreatic and biliary tract cancer was diagnosed and staged on the basis of ultrasonography, abdominal computed tomography, magnetic resonance imaging, ultrasound endoscopy, endoscopic retrograde cholangiopancreatography, positron emission tomography, cytological or histological examinations, and explorative laparotomy. NAC(RT) was administered to patients with borderline resectable pancreatic cancer as defined by National Comprehensive Cancer Network Guidelines Version 1/2012 [19]. NAC was administered to patients with biliary tract (hilar cholangiocarcinoma with arterial invasion, metastatic lymph nodes, or Bismuth type IV) or gallbladder cancer (plural metastatic lymph nodes or clinical T3–4 disease according to the tumor-node-metastasis classification of the International Union Against Cancer, 7th edition [20]).

### Neoadjuvant chemo(radio)therapy

The NAC(RT) regimen for patients with pancreatic or biliary tract cancer consisted of gemcitabine (1000 mg/m^2^ administered intravenously on days 8 and 15) plus tegafur/gimeracil/oteracil (60 mg/m^2^ administered orally on days 1–14). Patients with pancreatic cancer (*n* = 50) received two courses of GS followed by 30 Gy of radiation therapy. Patients with biliary tract cancer (*n* = 14) received three courses of GS.

### Evaluated factors

Age, sex, body mass index, body surface area, Eastern Cooperative Oncology Group performance status, and a history of smoking, cardiovascular disease, and diabetes mellitus before receiving NAC(RT)-GS were evaluated from clinical records. CI_max_, white blood cell count, ANC, lymphocyte count, platelet count, serum interleukin 6 level, C-reactive protein level, complement C3 titer, complement C4 titer, 50.0% hemolytic unit of complement, albumin level, prognostic nutritional index, serum carcinoembryonic antigen level, carbohydrate antigen 19-9 level, pancreatic cancer-associated antigen level, and s-pancreas-1 antigen level were also evaluated.

### Maximum chemiluminescence intensity

CI_max_ was assessed based on an in vitro reaction between peripheral neutrophils and endotoxin. An endotoxin activity assay was performed as described previously [21]. Fifty microliter samples of whole blood and appropriate controls were incubated in duplicate with saturating concentrations of an anti-lipid A immunoglobulin M antibody, and then stimulated with opsonized zymosan. The resulting respiratory burst was detected by a chemiluminometer (Autolumat LB953; Berthold Technologies GmbH & Co. KG, Bad Wildbad, Germany) as light released from the lumiphore luminol. The maximum stimulated response (termed as CI_max_ by Kiguchi et al. [18]) was measured using lipopolysaccharide (4.6 ng/mL) as the stimulant.

### Endpoints

The primary endpoint was the incidence of Grade ≥ 3 SN (ANC < 1000/mL) during NAC(RT)-GS. The observation period was from the first to the last day of NAC(RT)-GS. The secondary endpoint was the RDI of gemcitabine.

### Statistical analyses

Data are expressed as the median and range or number and percentage. Continuous variables were analyzed using the Mann–Whitney *U* test and categorical variables were analyzed using Chi square or Fisher’s exact test. Univariate and multivariate logistic regression analyses were performed to determine independent predictors of the incidence of SN during NAC(RT)-GS. Odds ratios and their 95.0% confidence intervals were calculated. Continuous variables were adjusted by dividing by the standard deviation and comparing to the odds ratio. Statistical analyses were conducted using Statistical Package for the Social Sciences for Windows (software version 23.0; SPSS Japan Inc., Tokyo, Japan). A *P* < 0.05 was considered statistically significant.

## Results

### Patient characteristics

The patient characteristics are summarized in Table 1. The median age was 71 (range 39–85) years. Thirty-six patients (56.3%) were male. All patients had an Eastern Cooperative Oncology Group performance status of 0–1. The general condition of the patients was satisfactory. Fifty patients (78.1%) had advanced pancreatic cancer. Fourteen patients (21.9%) had advanced biliary tract cancer. The median serum albumin levels were slightly lower, while the carbohydrate antigen 19-9 levels, pancreatic cancer-associated antigen levels, and s-pancreas-1 antigen levels were higher, than their normal ranges. SN was detected in 39 patients (60.9%), within a median of 15 (range 10–36) days from commencing NAC(RT)-GS. All instances of SN occurred during the chemotherapy period and not the radiotherapy period.

**Table 1:** Patient characteristics

Characteristic	Patients (*n* = 64)
Age (years), median (range)	71 (39–85)
Sex, *n* (%)	
M	36 (56.2)
F	28 (43.8)
ECOG PS (0–1), *n* (%)	64 (100.0)
BSA (m^2^), median (range)	1.6 (1.1–1.9)
BMI (kg/m^2^), median (range)	21.0 (14.3–29.8)
Type of cancer, *n* (%)	
Pancreatic	50 (78.1)
Biliary tract	14 (21.9)
Smoking history, *n* (%)	31 (48.4)
CVD, *n* (%)	8 (12.5)
DM, *n* (%)	26 (40.6)
Biliary drainage, *n* (%)	29 (45.3)
WBC (/µL), median (range)	5450 (3200–10500)
ANC (/µL), median (range)	3349 (1839–7603)
LC (/µL), median (range)	1360 (507–2789)
PC (× 10^3^/µL), median (range)	19.4 (10.7–33.3)
CI_max_ (RLU/s), median (range)	58,080 (7371–521,141)
CI_max_/neu, median (range)	15.8 (2.2–113.9)
IL-6 (pg/mL), median (range)	2.7 (0.9–63.7)
CRP (mg/dL), median (range)	0.15 (0.01–4.27)
Serum albumin (g/dL), median (range)	4.0 (2.6–5.0)
O-PNI, median (range)	46.4 (31.0–59.9)
CEA (ng/mL), median (range)	2.8 (0.9–32.3)
CA19-9 (U/mL), median (range)	109.5 (1.0–22,389.0)
DUPAN-2 (U/mL), median (range)	180.0 (17.0–57,000.0)
SPan-1 (U/mL), median (range)	53.0 (1.0–9700.0)

*ANC* absolute neutrophil count, *BMI* body mass index, *BSA* body surface area, *CA19-9* carbohydrate antigen 19-9, *CEA* carcinoembryonic antigen, *CI*_*max*_ maximum chemiluminescence intensity, *CRP* c-reactive protein, *CVD* cardiovascular disease, *DM* diabetes mellitus, *DUPAN-2* pancreatic cancer-associated antigen, *ECOG* Eastern Cooperative Oncology Group, *F* female, *IL-6* interleukin 6, *LC* lymphocyte count, *M* male, *O-PNI* Onodera’s prognostic nutritional index, *PC* platelet count, *PS* performance status, *RLU* relative light unit, *SPan-1* s-pancreas-1 antigen, *WBC* whole blood count

### Univariate analysis

The results of the univariate analysis are summarized in Table 2. CI_max_, ANC, interleukin 6 level, C-reactive protein level, complement C3 titer, and 50.0% hemolytic unit of complement before NAC(RT) were identified as significant factors. Conversely, no epidemiological, tumor-related, or nutritional factors were found to be significant.

**Table 2:** Univariate analysis of factors predicting severe neutropenia (SN) in patients with advanced pancreatic or biliary tract cancer

Factor	Patients		*P* value
	SN(+) (*n* = 39)	SN(−) (*n* = 25)	
Age (years), median (range)	69 (39–85)	73 (60–80)	0.210
Sex, *n* (%)			
M	20 (51.3)	16 (64.0)	
F	19 (48.7)	9 (36.0)	0.229
BSA (m^2^), median (range)	1.6 (1.1–1.9)	1.6 (1.2–1.9)	0.923
BMI (kg/m^2^), median (range)	21.4 (16.4–29.8)	20.8 (14.3–27.5)	0.591
Type of cancer, *n* (%)			
Pancreatic	30 (76.9)	20 (80.0)	
Biliary tract	9 (23.1)	5 (20.0)	0.513
Smoking history, *n* (%)	20 (51.3)	11 (44.0)	0.378
CVD, *n* (%)	3 (7.7)	5 (20.0)	0.144
DM, *n* (%)	14 (35.9)	12 (48.0)	0.241
Biliary drainage, *n* (%)	15 (38.5)	14 (56.0)	0.204
WBC (/µL), median (range)	5200 (3200–8900)	6200 (4000–10,500)	0.087
ANC (/µL), median (range)	3197 (1839–7387)	4222 (2035–7603)	0.017*
LC (/µL), median (range)	1314 (506–2789)	1,445 (568–2415)	0.778
PC (× 10^3^/µL), median (range)	18.1 (11.6–33.3)	21.0 (10.7–30.9)	0.299
CI_max_ (RLU/s), median (range)	46,739 (7370–156,539)	70,041 (34,831–521,140)	0.006*
CI_max_/ANC, median (range)	15.3 (2.2–40.4)	17.9 (8.9–113.9)	0.100
IL-6 (pg/mL), median (range)	2.3 (0.9–63.7)	3.9 (1.0–22.5)	0.014*
CRP (mg/dL), median (range)	0.11 (0.01–2.27)	0.39 (0.01–4.27)	0.028*
Serum C3 (mg/dL), median (range)	105.0 (74.0–139.0)	113.0 (79.0–177.0)	0.018*
Serum C4 (mg/dL), median (range)	28.0 (15.0–43.0)	28.0 (13.0–42.0)	0.534
CH50 (U/mL), median (range)	46.1 (31.5–61.5)	52.4 (36.3–91.8)	0.011*
Serum albumin (g/dL), median (range)	4.0 (2.6–5.0)	3.9 (2.8–4.9)	0.216
O-PNI, median (range)	47.0 (36.1–59.9)	45.2 (9.4–57.2)	0.470
CEA (ng/mL), median (range)	3.5 (0.8–220.8)	2.2 (0.9–32.3)	0.794
CA19-9 (U/mL), median (range)	101.0 (1.0–2,760.0)	35.5 (6.0–28,874.0)	0.659
DUPAN-2 (U/mL), median (range)	240.0 (25.0–57,000.0)	89.5 (25.0–18,000.0)	0.474
SPan-1 (U/mL), median (range)	47.0 (1.2–2000.0)	21.0 (3.3–21,000.0)	0.620
S1 RDI (%), median (range)	100.0 (30.0–100.0)	100.0 (33.0–100.0)	0.249
GEM RDI (%), median (range)	65.0 (30.0–100.0)	75.0 (33.0–100.0)	0.035*
Cholangitis during NACRT, *n* (%)	7 (15.9)	6 (23.1)	0.330

(+) positive, (−) negative, *ANC* absolute neutrophil count, *BMI* body mass index, *BSA* body surface area, *C3* complement C3, *C4* complement C4, *CA19-9* carbohydrate antigen 19-9, *CEA* carcinoembryonic antigen, *CH50* 50.0% hemolytic unit of complement, *CI*_*max*_ maximum chemiluminescence intensity, *CVD* cardiovascular disease, *DM* diabetes mellitus, *DUPAN-2* pancreatic cancer-associated antigen, *F* female, *GEM* gemcitabine, *IL-6* interleukin 6, *LC* lymphocyte count, *M* male, *NACRT* neoadjuvant chemoradiotherapy, *O-PNI* Onodera’s prognostic nutritional index, *PC* platelet count, *RDI* relative dose intensity, *RLU* relative light unit, *S1* tegafur/gimeracil/oteracil, *SPan-1* s-pancreas-1 antigen, *WBC* white blood cell count

*P < 0.05

### Multivariate analysis

The results of the multivariate analysis, which was performed using the six significant factors identified in the univariate analysis, are summarized in Table 3. Independent variables were selected using the simultaneous method for CI_max_ and stepwise methods for the remaining five factors (the criterion for adding a new variable was *P* < 0.05). CI_max_ was identified as a significant independent predictor of SN during NAC(RT)-GS (odds ratio: 0.248, 95.0% confidence interval 0.073–0.850; *P* = 0.026).

**Table 3:** Multivariate analysis of factors predicting severe neutropenia in patients with advanced pancreatic or biliary tract cancer

Factor	OR (95.0% CI)	*P* value	OR (95.0% CI)	*P* value
CI_max_ (/SD)	0.270 (0.100–0.724)	0.009*	0.248 (0.073–0.850)	0.026*
ANC (/SD)	0.517 (0.291–0.916)	0.024*	–	–
IL-6 (/SD)	0.822 (0.458–1.477)	0.513	–	–
CRP (/SD)	0.514 (0.266–0.994)	0.048*	–	–
Serum C3 (/SD)	0.738 (0.461–1.183)	0.207	–	–
CH50 (/SD)	0.662 (0.396–1.105)	0.114	–	–

*ANC* absolute neutrophil count, *C3* complement C3, *CH50* 50.0% hemolytic unit of complement, *CI* confidence interval, *CI*_*max*_ maximum chemiluminescence intensity, *CRP* c-reactive protein, *IL-6* interleukin 6, *OR* odds ratio, *SD* standard deviation

*P < 0.05

### Prediction ability

**Fig. 1:**
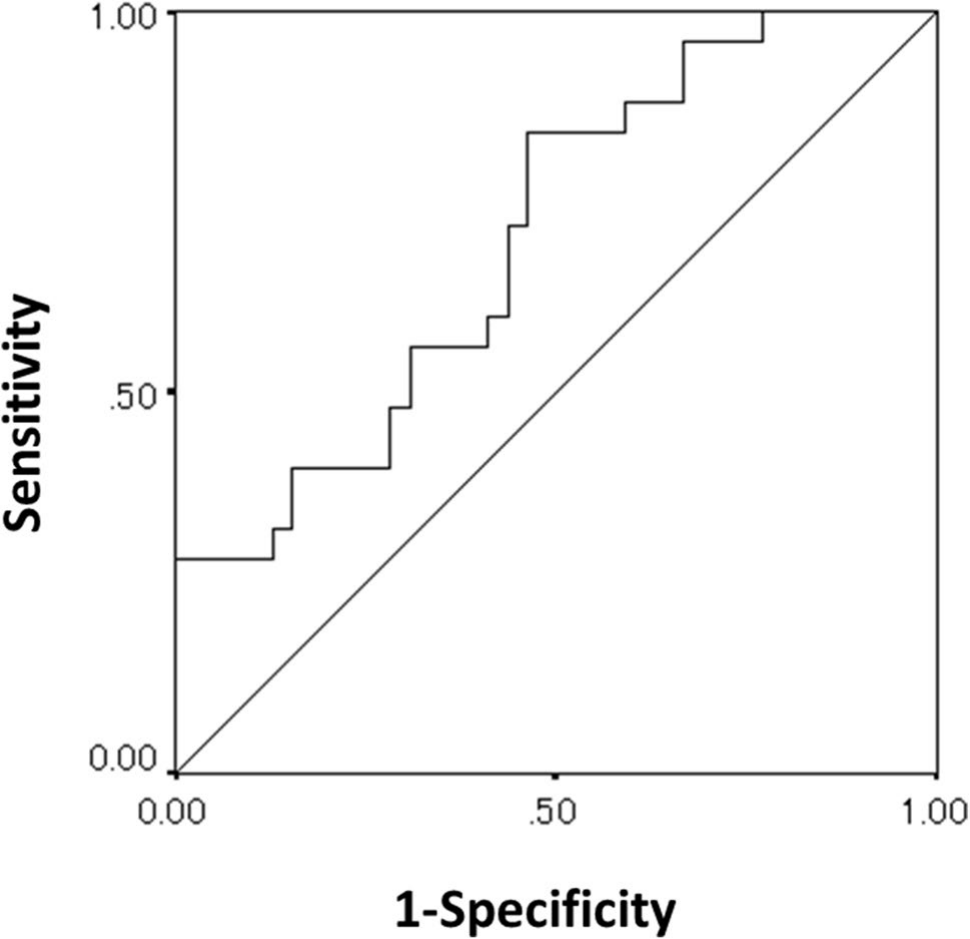
Receiver operating characteristic curve of the maximum chemiluminescence intensity for predicting severe neutropenia in patients with advanced pancreatic or biliary tract cancer

The area under the receiver operating characteristic curve of the incidence of SN predicted by CI_max_ was 0.704 (Fig. 1). The optimal threshold for the CI_max_ was 46,000 RLU/s. Applying this cutoff, the sensitivity and specificity were 46.2% and 80.0%, respectively. The majority of patients (*n* = 18; 81.8%) with a low CI_max_ before NAC(RT) experienced SN during NAC(RT)-GS.

### Relative dose intensity of gemcitabine

The RDI of gemcitabine in patients with SN was lower than in those without SN (Table 2). At CI_max_ cutoff of 46,000 RLU/s, the median RDI of gemcitabine was significantly lower in the low CI_max_ group than in the high CI_max_ group (65.0% vs. 75.0%, respectively; *P* = 0.014).

## Discussion

Gemcitabine has a wide spectrum of anticancer activities with few non-hematological adverse events [22]. However, the GEST study [9] showed that SN had occurred in 62.2% of patients with locally advanced and metastatic pancreatic cancer in Japan and Taiwan. Consistent with the GEST study [9], the incidence of SN in our study was 60.9%. Patients who develop SN during NAC require serial dose reductions until a tolerable dose is reached. In this study, the RDI of gemcitabine was significantly lower in patients who developed SN.

Studies [10–16] have shown epidemiological (age, sex, or a prior history), tumor-related, nutritional, and inflammatory statuses to be risk factors for SN. However, in relation to gemcitabine-based chemotherapy, risk factors for SN include a low ANC, a low white blood cell count, a low carbohydrate antigen 19-9 level, and no prior history of smoking [17, 23, 24]. In this study, no association was identified between epidemiological, tumor-related, or nutritional factors and the incidence of SN during NAC(RT)-GS.

In the present study, logistic regression analysis identified CI_max_ before NAC(RT) as an independent predictor of SN during NAC(RT)-GS. At an optimal cutoff value of 46,000 RLU/s, the specificity of the CI_max_ for predicting SN was 80.0%. The positive predictive value was 81.8%.

In patients with lipopolysaccharide-induced sepsis, the oxidative burst is significantly diminished in non-survivors compared to survivors [25]. Reduced oxidative activity may be associated with immune dysfunction and high mortality [25, 26]. Kiguchi et al. [18] reported that CI_max_ before commencing treatment is indicative of the maximum neutrophil activity in whole blood and is highly predictive of mortality in patients with sepsis. CI_max_ reflects neutrophil vitality or fatigue. Therefore, we hypothesized that CI_max_ may be a predictor of SN during NAC.

In this study, univariate analysis identified a low CI_max_, a low ANC, a low interleukin 6 level, a low C-reactive protein level, a low complement C3 titer, and a low 50.0% hemolytic unit of complement as risk factors for SN. In advanced cancer, chronic inflammation caused by an elevation in inflammatory cytokines leads to tumor progression [27], and activates neutrophil functions, such as the expression of adhesion molecules, phagocytosis, and the production of reactive oxygen species [28]. CI_max_ reflects the reactive oxygen species production of neutrophils. Therefore, a low CI_max_ indicates the inhibition of these inflammatory reactions. Recently, it has been reported [29, 30] that single nucleotide polymorphisms (SNPs) determine the individual components and/or the total white blood cell count. Furthermore, SNPs may correlate with the incidence of chemotherapy-induced neutropenia. Patients with SN may have a low baseline CI_max_, resulting in the overall inhibition of inflammatory responses due to unknown SNPs, which result in lower neutrophil counts and reduced neutrophil activity. CI_max_ may be a surrogate marker of not only predicting the incidence of SN, but also the patient’s overall immune function.

Measurement of CI_max_ is quick and convenient for clinical use. If CI_max_ could be adopted in daily clinical practice then patients at risk of developing SN may be given prompt and appropriate treatment. Classifying patients into different risk groups based on their CI_max_ may also help physicians choose the optimal treatment strategy. For instance, patients at a high risk of SN can be started with a reduced dose of GS and have their ANC strictly monitored by frequent blood tests. If patients are informed of their risk of neutropenia in advance, they can pay more attention to their own condition during treatment.

**Fig. 2:**
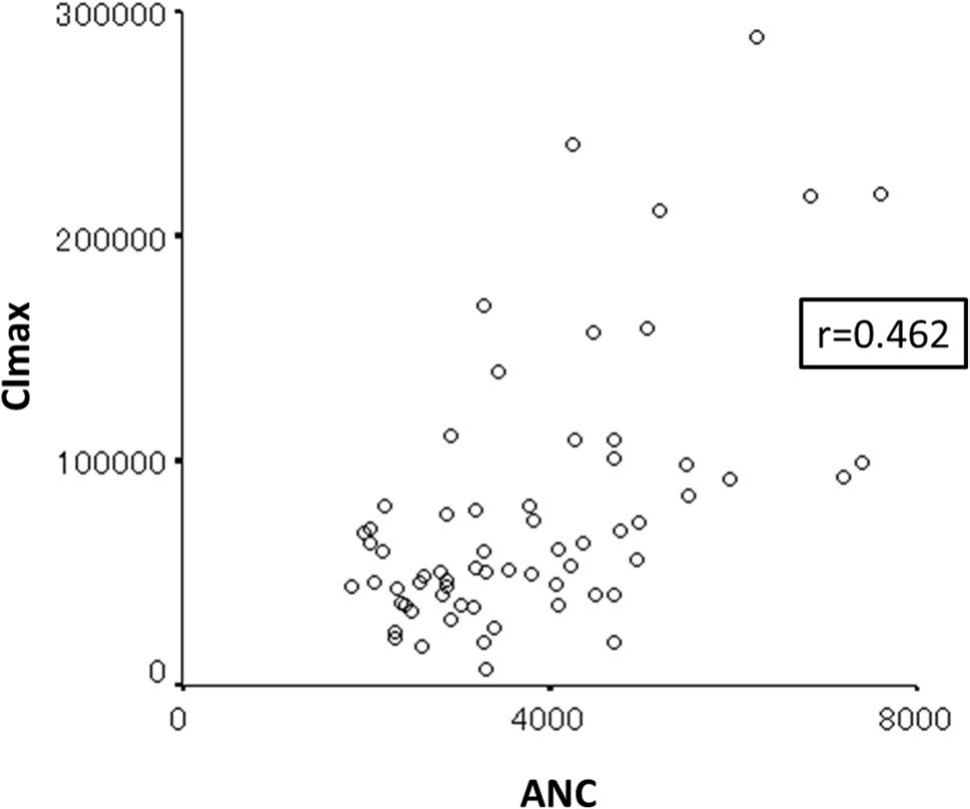
Correlation between the maximum chemiluminescence intensity (CI_max_) and absolute neutrophil count (ANC)

There is the potential that SN may arise exclusively from a low baseline ANC due to the inhibition of inflammation or neutrophil lowering SNPs. In other words, a low baseline ANC alone may be sufficient to cause to SN. However, we determined that the correlation coefficient between CI_max_ and ANC was 0.462 (Fig. 2). Hence, CI_max_ and ANC are independent factors. The multivariate analysis showed CI_max_ to be a better predictive factor, representing the conclusive status of neutrophils.

SN is associated with the long-term prognosis of patients with various types of cancer, including breast [31, 32], small cell lung [33, 34], gastric [35], colorectal [36, 37], ovarian [38], and pancreatic [39, 40]. The long-term prognosis may be associated with SN and CI_max_, though this was not evaluated in this study. We are currently conducting a prospective cohort study focusing on the long-term outcomes of patients with advanced pancreatic cancer.

There are two limitations of this study. The first is that only a limited number of cases from a single institution were available at the time of conducting the study and the second is that only NAC(RT)-GS cases of advanced pancreatic or biliary tract cancer were analyzed. This paper only reports preliminary results. However, we believe that our findings are of interest. Multicenter studies involving greater number of patients with varying types of cancer and NAC regimens are needed to evaluate whether a low CI_max_ is a useful marker for predicting SN.

In conclusion, our findings suggest that patients with advanced pancreatic or biliary tract cancer with a low CI_max_ who are receiving NAC(RT) are at a high risk of developing SN during NAC(RT)-GS. Further studies with larger sample sizes are needed to validate our findings with other regimens (e.g., FOLFIRINOX and gemcitabine plus nanoparticle albumin-bound paclitaxel) and to confirm the associations between CI_max_, SN, SNPs, and long-term survival.
